# Implementing Web-Based Interventions in HIV Primary Care Clinics: Pilot Implementation Evaluation of Positive Health Check

**DOI:** 10.2196/10688

**Published:** 2019-04-18

**Authors:** Camilla Harshbarger, Olivia Burrus, Brittany A Zulkiewicz, Alexa M Ortiz, Carla A Galindo, Bryan R Garner, Robert D Furberg, Megan A Lewis

**Affiliations:** 1 Division of HIV/AIDS Prevention Centers for Disease Control and Prevention Atlanta, GA United States; 2 Center for Communication Science RTI International Durham, NC United States; 3 Digital Health and Clinical Informatics RTI International Durham, NC United States; 4 Behavioral and Urban Health Program RTI International Durham, NC United States

**Keywords:** internet, patient care, attitudes, vulnerable populations, public health practice

## Abstract

**Background:**

Web-based interventions can help people living with HIV achieve better clinical outcomes and behaviors, but integrating them into clinical practice remains challenging. There is a gap in understanding the feasibility of implementing these interventions in HIV clinic settings from the clinicians’ perspective.

**Objective:**

The goal of the research was to determine whether Positive Health Check (PHC)—a Web-based, tailored video counseling tool focused on increasing patient adherence and retention in care and reducing HIV risk among HIV-positive patients—was acceptable, appropriate, and feasible for HIV primary care clinic staff to implement in clinic workflows.

**Methods:**

A multiple-case study design was used to evaluate the pilot implementation. Four primary care clinics located in the southeastern United States implemented PHC over a 1-month period. Nine clinic staff across the clinics participated in structured interviews before, during, and after the implementation. In total, 54 interviews were conducted. We used a framework analysis approach to code the data and identify themes related to implementation outcomes, including acceptability, appropriateness, and feasibility. We also analyzed patient intervention use metrics (n=104) to quantify patient intervention completion rates (n=68).

**Results:**

Overall, clinicians viewed PHC as acceptable and appropriate. Themes that emerged related to these implementation outcomes include the ability for PHC to increase provider-patient communication and its ability to engage patients due to the tailored and interactive design. While generally feasible to implement, challenges to the clinic workflow and physical environment were areas that clinics needed to manage to make PHC work in their clinics.

**Conclusions:**

Findings from this pilot implementation suggest that clinical staff viewed PHC as acceptable and appropriate, especially as more patients used the intervention over the pilot period. Feasibility of implementation was challenging in some cases, and lessons learned from this pilot implementation can provide information for larger scale tests of the intervention that include assessment of both implementation outcomes and clinical outcomes.

## Introduction

Interventions are being developed for clinical settings to retain and support patients in HIV care [1,2]. However, the clinical context presents barriers to implementing new HIV retention and adherence interventions [3-7]. These barriers need to be addressed before widespread adoption of clinic-based interventions can occur [8].

Computer-based HIV adherence interventions appear to be feasible and acceptable from the patient perspective [9], but they have not been studied from the perspective of the clinic stakeholder implementing such interventions. The benefit of understanding implementation is that strategies can be designed to facilitate integration of evidenced-based interventions into care. Positive Health Check (PHC) is a brief, interactive Web-based video counseling tool to reduce HIV transmission and improve health outcomes for people living with HIV (PLH). The video tool was developed based on evidence that computer-based counseling tools can reduce sexual risk behaviors and improve antiretroviral therapy adherence [1,2,10-13] and viral load suppression [12]. PHC is grounded in the information-motivation-behavior model [14], assuming that providing information and building motivation and skills for medication adherence, appointment keeping, and other behaviors will result in PLH correctly practicing behaviors needed to manage HIV and improve health outcomes. We used principles of motivational interviewing [15] to guide the scripting of empathic language. The transtheoretical model [16] informed tailoring scripts, for example, around the extent to which individual HIV patients choose to interact with PHC intervention modules, ask questions of their provider, or select and practice behavioral strategies.

The making of PHC was a dynamic 3-year endeavor involving many stakeholders. The goal was to build a Web-based intervention that would support and be adopted by HIV clinics, be easily updated and scaled up, and improve patient health outcomes. We engaged a user-centered approach and gathered detailed and iterative feedback on the many steps taken to design, develop, and implement PHC from prospective HIV-patient users and HIV providers, including both implementers and gatekeepers. This process is described in Harshbarger et al [17]. To film, manage, program, use feedback, and fine-tune PHC from the database of 700 video clips, the PHC team employed an agile development process [18]. We worked with many contributors, including infectious disease researchers, app developer, videographer, graphic artist, and closed captioning specialist.

The purpose of the pilot implementation evaluation of PHC we describe here was to understand the barriers and facilitators to implementing the intervention in busy clinic settings. Specifically, we were interested in how clinic stakeholders managed the implementation. We examined their perceptions of implementation outcomes including (1) appropriateness (ie, the perceived fit, relevance, or compatibility of PHC to support clinic efforts to improve patient viral load suppression and well-being), (2) acceptability (ie, the perception among implementation stakeholders that PHC is agreeable, palatable, or satisfactory and supports the mission of the HIV clinics), and (3) feasibility (ie, the extent to which clinic staff can successfully administer or use PHC within the busy workflow of HIV clinic settings) [19,20]. Relying on data from the tool itself, we also aimed to determine if staff could provide patients with sufficient time in waiting rooms to complete the tool. This paper aims to address a gap in the literature to better understand staffer implementation of Web-based interventions in their complex clinical settings and workflows [21].

## Methods

### Positive Health Check Intervention and Pilot Implementation Evaluation Procedures

**Figure 1:**
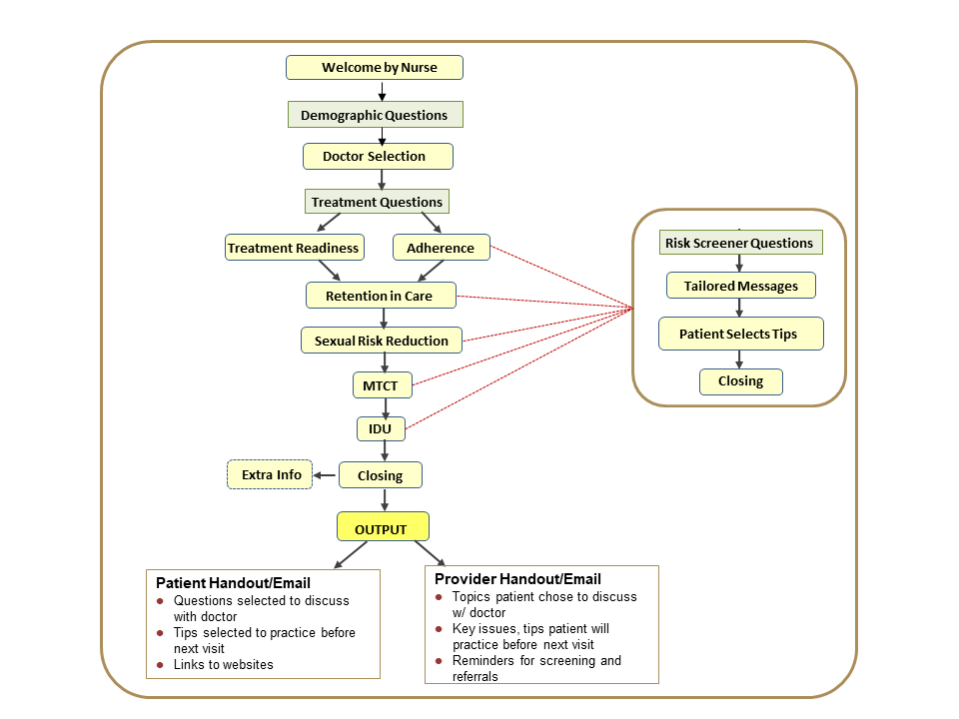
Positive Health Check patient experience flow diagram. IDU: injection drug use; MTCT: mother-to-child transmission.

An overview of PHC is shown in Figure 1. PHC is introduced to patients by a designated clinic staffer, referred to as an onboarder, who offers an eligible patient in the waiting room the opportunity to use the Web-based intervention. The onboarder accesses the PHC clinic Web application (CWA) to generate a user ID and password. Based on responses to questions about clinic attendance, medication use, and HIV risk behaviors, each patient watches individually tailored videos addressing HIV treatment readiness, antiretroviral therapy adherence, retention in HIV medical care, sexual risk reduction, prevention of mother-to-child transmission, and safer injection drug use practices. Patients can select questions to ask their clinic doctor during their scheduled appointment, and they are provided behavioral strategies, called tips, to practice. A patient handout featuring this information is automatically printed and delivered to the patient, and a truncated version is delivered to the provider at the request of the patient. At the end of the intervention, patients can also click on “Extra Info” to view supporting resource materials. The onboarder uses the CWA to track process data, including the number of patients who logged on, completed the intervention, or requested that a link to PHC be sent to their private email. PHC does not collect any personal identifying information or patient data, and no email addresses are stored.

### Study Design

This implementation evaluation pilot was conducted from May to July 2015, and each of the four participating clinics implemented PHC for 1 month during that period. A multiple case-study design [22] was used to gather process evaluation data from clinic staff to examine and describe the contextual and implementation issues that might help other clinics adopting PHC prepare for implementation [23]. We also analyzed de-identified tool use data to understand patient navigation, use, and completion of the intervention.

### Clinic Eligibility

Four HIV clinics were located in rural, urban, and suburban areas across the southeastern United States. All clinics were required to (1) provide primary HIV care to at least 200 HIV-positive patients annually; (2) use the PHC CWA on a secure, networked Windows desktop, workstation, or server; and (3) have broadband internet access that supports wireless access for iPad and Android devices. The characteristics of the four participating clinics are summarized in Table 1.

### Patient Eligibility

HIV-positive English-speaking patients who were at least 18 years old were eligible to use the intervention during the pilot implementation evaluation. Onboarders invited patients to use PHC after they checked in to see their provider. One clinic contacted patients by telephone before appointments and issued invitations.

### Preparing Clinics for Implementation

We provided each clinic with three tablets equipped with high-impact protective cases, privacy screens, and headphones for individual patient use. Additionally, we trained clinic staff how to use PHC and the CWA to onboard patients and generate summary reports. Staff also received user guides covering all aspects of intervention implementation.

### Data Collection

We collected feedback from clinical staff (N=9), including the intervention onboarder and one primary care provider, at each clinic. At one site, an additional HIV primary care provider participated. We also collected data from the CWA to understand patient use of the intervention (eg, whether the patient completed the intervention; Table 2).

Pairs of interviewers conducted a series of semistructured interviews with each key informant. Each informant participated in 6 interviews: 1 face-to-face preimplementation interview, 4 weekly telephone interviews, and 1 telephone wrap-up interview, for a total of 54 interviews across all 4 clinics. Preimplementation questions focused primarily on perceptions of clinic preparedness and satisfaction with trainings and training materials. During the weekly interviews, we asked participants questions related to the implementation of PHC, including barriers and facilitators; contextual factors; and acceptability, appropriateness, and feasibility of the intervention. For example, we asked, “What types of patients do you think will most benefit from the intervention?” (appropriateness); “As a provider, what do you like the most/least about Positive Health Check?” (acceptability); “Describe to me how the tool is typically incorporated into your workflow, from the beginning to the end of a patient’s appointment” (appropriateness); “What aspects of your clinic environment do you think were an issue for how PHC was implemented? Why?” (feasibility); and “How, if at all, has implementing Positive Health Check affected the workflow of the doctors at your clinic?” (acceptability/feasibility). Wrap-up interviews touched on these same topics and asked for suggested changes to the intervention and perceptions of sustainability. Development and pilot implementation of the tool was approved as nonresearch by the Office of the Associate Director for Science in the Division of HIV/AIDS Prevention at the US Centers for Disease Control and Prevention (CDC).

### Analysis

Interview notes were entered into NVivo 10.0 (QSR International Pty Ltd). Notes were tagged by clinic, key informant type, and implementation phase (preimplementation, implementation weeks 1 through 4, and postimplementation). Notes were checked against audio recordings as needed for clarification and to ensure the accuracy of direct quotes used in reporting. A team of four trained staff coded the interviews using a topical codebook that operationalized concepts from the interviews. Concepts included barriers and facilitators, contextual factors, acceptability, appropriateness, feasibility, and sustainability.

**Table 1:** Characteristics of HIV primary care clinics in the pilot implementation evaluation (percentages may not add to 100% because of rounding).

Characteristics				Clinic A^a^		Clinic B^a^		Clinic C		Clinic D
**Clinic demographics**										
	Type of service area		Rural		Urban		Suburban		Urban/suburban	
	Type of clinic		Nonprofit clinic		Ambulatory clinic, multispecialty practice, nurse-managed clinic		Ambulatory clinic, public hospital, academic medical center		Ambulatory clinic, primary care practice, specialty care practice	
	Patient visits per year		1000		800		4617		7400	
	Patient visits per day, average		10		15-20		50		8	
**Patient demographics**										
	HIV-positive patients, n (%)		257 (100)		140 (100)		1927 (90)^b^		1166 (100)	
	**Sex, n (%)**									
		Male		(60)		(60)		1360 (70.6)		825 (70.8)
		Female		(40)		(40)		557 (28.9)		332 (28.5)
		Transgender		(0.1)		Unknown		10 (0.1)		Unknown
	**Race, n (%)**									
		White		(13)		(39)		610 (31.7)		301 (25.8)
		Black or African American		(87)		(60)		1133 (58.8)		824 (70.7)
		American Indian or Alaska Native		(0)		(0)		23 (1.2)		1 (0.1)
		Asian		(0)		(1.0)		15 (0.8)		6 (0.5)
		Other/unknown		(0)		(0)		145 (7.4)		34 (2.9)
	**Ethnicity, n (%)**									
		Hispanic or Latino		(2)		(15)		140 (7.3)		117 (10.0)
		Not Hispanic or Latino		(98)		(85)		1787 (92.7)		1049 (90.0)
	**Age (years), n (%)**									
		<13		(0)		(4)		0 (0)		0 (0)^c^
		13-24		(4)		(96)		81 (4.2)		14 (1.2)^c^
		25-44		(31)		(0)		727 (37.7)		280 (24.0)^c^
		45-64		(59)		(0)		1028 (53.3)		672 (57.6)^c^
		>65		(6)		(0)		91 (4.7)		199 (17.1)^c^

^a^Clinics A and B reported demographics in rounded percentages only (except for number of HIV-positive patients); actual values are not available.

^b^Clinic C was not an HIV exclusive clinic; 90% of their patients were HIV positive.

^c^Age ranges for Clinic D were reported in different ranges than other clinics: <18, 18-21, 22-35, 36-55, and 56-80 years.

**Table 2:** Use information generated from the clinic Web application for Positive Health Check.

Category	Clinic A		Clinic B		Clinic C			Clinic D	
	n (%)	N	n (%)	N	n (%)	N	n (%)		N
Total approached^a^	17 (100)	—	16 (100)	—	91 (100)	—	21 (100)		—
Declined^b^	1 (6)	17	1 (6)	16	34 (37)	91	5 (24)		21
Onboarded^c^	16 (94)	17	15 (94)	16	57 (63)	91	16 (76)		21
Complete^d^	15 (94)	16	14 (93)	15	29 (51)	57	10 (63)		16
Incomplete^e^	1 (6)	16	1 (7)	15	22 (39)	57	5 (31)		16
Assigned^f^	0 (0)	16	0 (0)	15	6 (11)	57	0 (0)		16
Refused^g^	0 (0)	16	0 (0)	15	0 (0)	57	1 (6)		16
Patient handouts generated^h^	11 (73)	15	8 (57)	14	10 (35)	29	8 (80)		10
Patient handouts delivered^i^	10 (91)	11	5 (63)	8	5 (50)	10	8 (100)		8
Provider handouts generated^j^	7 (47)	15	4 (29)	14	6 (21)	29	3 (30)		10
Provider handouts delivered^k^	5 (71)	7	2 (50)	4	3 (50)	6	0 (0)		3

^a^Onboarder asked the patient whether he or she wanted to use Positive Health Check (PHC).

^b^Patient declined to use PHC. Percentages calculated based on N approached.

^c^Patient agreed to use PHC and was assigned a study ID. Onboarded percentages calculated based on N approached.

^d^Patient completed the entirety of the PHC tool. Percentages calculated based on n onboarded.

^e^Patient did not complete the PHC tool. Handouts were not generated. Percentages for incomplete calculated based on n onboarded.

^f^Patient agreed to participate and was assigned a study ID but did not log in to the PHC tool. Percentages calculated based on n onboarded.

^g^Patient agreed to participate, was assigned a study ID, and logged in to the PHC tool but did not accept at the consent screen. Refused percentages calculated based on n onboarded.

^h^Handouts were generated only for patients who completed the PHC tool and selected tips and/or questions for their providers. Percentages calculated based on n complete.

^i^Patient handouts were delivered directly to the patient. Percentages calculated based on n handouts generated.

^j^Provider handouts were generated and printed only for patients who completed the PHC tool, selected tips and/or questions for their providers, and agreed to share the handout with their provider.

^k^Provider handouts were delivered in person to the patient’s provider by the onboarder, to the patient’s medical chart, or on the exam room door, depending on the clinic’s implementation protocol.

We conducted two rounds of test coding to establish consistency between coders.

We then used the framework analysis method [24] to identify themes within and across sites, generating theme-based charts to organize the data by code, clinic staff position, site, and time point. To complete the framework analysis, team members completed one round of thematic analysis on the same topical code to ensure consistent interpretation of the data. Discrepancies were reconciled, and team members then worked independently to identify themes in each code. A theme was deemed important when multiple interviewees echoed the same idea within the same site, across sites, and over time. Quotes from interview participants illustrating themes are included in Results. We summarize descriptive statistics about tool use generated from the CWA in Table 2.

## Results

### Patient Consent and Completion Rates

A total of 145 patients were approached to use PHC across the 4 clinics, and 104 (71.7%) patients agreed to participate. Although 68 (65.4%) of those 104 patients completed PHC, 29 (27.9%) did not complete the intervention, with Clinics C and D having particularly high patient incompletion rates of 39% (22/57) and 31% (5/16), respectively.

### Acceptability and Appropriateness

#### Facilitating Provider-Patient Communication

We asked key informants to what extent clinicians perceived that PHC was acceptable and appropriate and what factors shaped these perceptions. Overall, clinicians reported that the intervention was acceptable and expressed support for its use and enthusiasm about its potential to support patients and providers.

This tool gave me an opportunity to understand that I probably need to do a better job of communicating with patients. In retrospect, the tool is good for the provider and if they look at what their patient’s concerns are and think it may change or may enhance the conversation they have with the patient.

Provider, Clinic D

Typically patients are so overwhelmed with their diagnosis that the tool could really help them break things down and see the information in a different way.

Provider, Clinic C

Clinicians at each of the four sites indicated that providers also would benefit from PHC because it supports interactions during the clinic visit, as facilitated largely by the tailored handouts. For example, several providers said that the handouts empowered patients to ask questions and identify information gaps that needed to be addressed. The handouts also gave providers a starting point for discussions with patients. In one case, the handouts led a patient to reveal an undisclosed sexually transmitted infection. A provider in Clinic A said, “I did use the handouts to see what their concerns were or what they wanted to know more about, and that changed my conversation.”

Providers at two clinics expanded their notions of who would benefit from the intervention over the course of the implementation period. One saw the potential benefits expand by adapting the intervention for loved ones and by conducting group sessions. Initially, developers perceived PHC as a way to support the patient when interacting with their provider. Yet over the course of implementation, providers in three clinics increasingly viewed PHC as helping them understand patient concerns. One provider learned she had been inadequately addressing medication adherence with her patients. Before implementation, this provider stated the following:

It is a great tool for patients, and also helps providers who don’t necessarily take the time to have conversations with patients that they should have. This may be the way for patients to get the information to start the conversation.

Provider, Clinic A

After implementation concluded, this provider was asked if her impression had changed.

I still think the tool is great for patients to initiate a conversation. I still think this is a good way for patients to open up. Now, I think it’s actually an opportunity for providers to be a little more interactive and to dig a little deeper in conversations with the patient.

Provider, Clinic A

#### Engaging Functionality and Interactive Design

At each of the four sites, clinicians and administrators reported PHC was an appropriate and acceptable intervention for helping PLH. In particular, the clinics liked the interactive components, such as patient selection of doctor and presentation of information in audio and video formats; the tailored messages based on patient responses; and information being presented in a clear and concise manner.

What I really liked the most about the tool was the fact the patient could select who they wanted to hear... If they wanted to have a female provider, if they wanted to have a provider of color, if they wanted to have a male versus a female...

Provider, Clinic A

These are important intervention features because electronic, tailored, and interactive interventions have been shown to be effective as they provide more appropriate information to patients, compared with interventions that are not tailored [25]. However, we also noted concerns about the length of PHC (at two of the clinics); ability to deliver the patient and provider handouts; and ease of navigation, with the exception of password generation.

#### Challenges to Acceptability

Relatively low computer literacy diminished the extent to which PHC was deemed to be appropriate for the population served by one rural clinic. Because of provider concerns about patient literacy in general and computer literacy specifically, during the first 2 weeks of implementation in this clinic, PHC was offered only to patients who were able to use the intervention without assistance. PHC was then offered to all patients during the last 2 weeks of implementation. At this point, it became clear that patients with lower computer literacy required more assistance, primarily due to the complexity of password generation, which required more of the onboarder’s time:

This [the tool] was very easy to use, but in rural areas like these many patients have no experience with the computer, at all. Many homes here don’t have Internet access...I think for people who don’t have any experience with computers, that they may not have really understood how the tool can be used...

Provider, Clinic A

### Feasibility

We also asked clinicians whether it was feasible to implement PHC as intended and what factors affected feasibility. Their responses revealed two main themes: clinic workflow and physical environment.

#### Clinic Workflow

Several factors related to clinic workflow presented implementation barriers. Respondents reported the challenge of scheduling patients to complete PHC without compromising tightly managed clinic workflows. Onboarders at Clinics A and B mentioned that PHC added 15 to 20 minutes to the time that patients spent in the clinic before seeing the provider, which caused delays. Additionally, onboarded patients were often interrupted to attend their provider appointments. Clinics C and D reported that clinic workflow processes interrupted patients engaging with the intervention. At Clinic C, patients were often called back to their appointment before the onboarder could deliver the handouts to the patient or provider. The onboarder from Clinic B described the experience fitting PHC into clinic workflow: “Possibly to try to get them [the patients] when they first come in the building as opposed to waiting for them to come into...the waiting room so they can view it [the tool] out there.”

Three of the clinics onboarded patients into the intervention before their appointments. Two of these clinics had patients complete the intervention in exam rooms, and the third clinic had patients complete it in the waiting room, with the option of finishing in the exam room. Conversely, the fourth clinic onboarded patients after their appointments, in a room designated specifically for intervention use, which key informants said was arranged because of possible workflow disruptions if delivered before the visit.

Sometimes patients can do the tool before they go back, but that’s if they are pretty early, not if they are right on time or late.

Onboarder, Clinic A

It would be much better if the clinic could have gotten the patient to use the tool before the visit... But the patients would need to come in early for their appointments.

Onboarder, Clinic C

#### Physical Environment

The clinic physical environments also posed barriers that affected implementation feasibility. Two clinics were challenged by finding private space for patients to complete the intervention. At one clinic where patients were completing the tool in exam rooms, there were not enough rooms on particularly busy days.

There were some cases during implementation where there was no space to complete the tool. If there was anything that hindered use of the tool, it would be the fact that it was very busy and there was overflow of patients.

Onboarder, Clinic C

Another challenge for three clinics was inconvenient locations for picking up handouts from the clinic fax printers. The location of the fax machine at the clinics, coupled with printing delays, led to handouts not being delivered consistently to patients or providers. To address some of these issues during implementation, we substituted the fax machines with wireless printers.

Handout delivery methods and success rates differed across clinics. Three clinics delivered handouts to patients in exam rooms, and this approach worked relatively well for two of the clinics, where they delivered 91% (10/11) and 63% (5/8) of handouts; however, it posed challenges for the third, where they delivered only 50% (5/10) of handouts. In the fourth clinic, the printer was in the same room where patients completed the tool; consequently, they typically would retrieve their own handouts (8/8, 100%) when generated. These findings suggest that patient handout delivery is feasible, but delivery methods call for refinement.

They would finish it [PHC] right before the provider came in, and once the provider is in there, I can’t give either patient or provider the handouts.

Onboarder, Clinic A

## Discussion

### Principal Findings

This pilot implementation evaluation demonstrated that in four clinics, HIV providers reported that the Web-based video tool PHC is an acceptable and appropriate intervention to supplement the support clinicians currently provide patients. They reported that PHC presents information in new ways that could strengthen patient-provider communication and help patients manage HIV. Clinicians indicated that provider handouts were useful because they increased their ability to address patients’ highlighted issues.

Feasibility, or the ability to embed PHC in clinic workflow without disruption, proved to be more complex. PHC onboarders had to gain the support of other clinic staffers, manage scheduling patient tool use and the performance of digital tablets and Wi-Fi printers, assign patients passwords to log into the tool, and deliver PHC-generated handouts to patients and providers prior to scheduled appointments. Two clinics (A and B) facilitated patients’ completion of the tool at relatively high rates (approximately 93%), but they reported that implementation increased patient wait time by 15 to 20 minutes and users were sometimes interrupted in order to minimize delays in workflow. Two clinics (C and D) reported more struggles with implementation, as reflected in the lower tool completion rates (51% and 63%, respectively). These clinics reported that PHC users were often interrupted in order to attend their provider appointment. To solve this problem, Clinic C started onboarding patients after their provider appointment, which undermined timely use of the patient and provider handouts.

PHC onboarders experienced difficulty in delivering patient and provider handouts as demonstrated by inconsistent delivery rates across clinics. The delivery of the provider handout was especially problematic due to the short amount of time between PHC completion and the start of the appointment. Onboarders had more success with the delivery of the patient handout due to the physical accessibility of the patient. We addressed two of the unanticipated barriers that slowed implementation efforts. The first was simplifying patients’ overly complex passwords requirements. The second was finding the correct technology to print patient handouts; consequently, during the pilot we provided wireless printers instead of fax printers.

Clinic preparation to implement digital interventions requires extensive strategizing, trial and error, and coordination across many clinic staff. We believe that the 1-month time frame was an insufficient period of time for clinics to practice and finely hone these preimplementation strategies. In addition, there was insufficient time to pilot offsite PHC features that can alleviate some of the described barriers to implementation, where patients can finish the tool at home or request that their handout be emailed to them.

Importantly, qualitative interview data show that clinic staffers presented solutions to many of the noted implementation barriers. These suggestions that can inform future PHC implementation include simplifying overly complex password requirements, meeting patients early in hallways by waiting rooms or asking patients to come to clinic early in order to engage with PHC. One suggestion requires more flexibility than was practical for the pilot—administering PHC after provider appointments where the handouts could be delivered at the next clinic appointment or sent to the user’s email address.

Aspects of the intervention design that resonated with the clinic staff included the interactive video format presenting information tailored to each user. These are important intervention features: electronic, tailored, and interactive interventions have been shown to be effective because they provide more appropriate information to patients compared with interventions that are not tailored [25].

This study underscores the concrete challenges of implementing digital interventions in complex and dynamic clinic environments, even when clinic stakeholders describe potential advantages and endorse the intervention. Pilot testing is critical to generate feedback from clinical stakeholders and produce implementation outcomes that will inform future implementation strategies in clinics.

### Limitations

Several limitations pertain to this implementation pilot. First, we were able to include only the viewpoints of select key clinic staff at each site. Future assessments that rely on clinic staff should include a larger number over a longer period to better understand their viewpoints on implementation. Second, patients were engaged in piloting the intervention; however, we did not obtain user feedback on experience and satisfaction, which would be critical to inform future efforts to implement Web-based interventions. Finally, although the four participating clinics varied in their type of service area and population base, PHC acceptability, appropriateness, and feasibility in other large urban clinics or those with extremely low resources require further study.

Despite these limitations, this pilot shows promise for the implementation of Web-based interventions like PHC. This work contributes to our understanding of clinic environments and strategies that support intervention implementation. Clinical settings are governed by complex workflow procedures and the need to follow regulatory guidelines and professional association best practices [26,27]. To facilitate the implementation of Web-based interventions that improve patient outcomes in clinical settings, clinicians need to address organizational workflow issues [28-32] and determine how these interventions can become a best practice. To close the gap in the HIV continuum of care for vulnerable populations, it is vital to understand and systematically study the implementation of Web-based interventions in clinical settings.

## Abbreviations

CDC: US Centers for Disease Control and Prevention
CWA: clinic Web application
PHC: Positive Health Check
PLH: people living with HIV

## References

[1] Fisher JD, Amico KR, Fisher WA, Cornman DH, Shuper PA, Trayling C, Redding C, Barta W, Lemieux AF, Altice FL, Dieckhaus K, Friedland G (2011). Computer-based intervention in HIV clinical care setting improves antiretroviral adherence: the LifeWindows Project. AIDS Behav.

[2] Gilbert P, Ciccarone D, Gansky SA, Bangsberg DR, Clanon K, McPhee SJ, Calderón SH, Bogetz A, Gerbert B (2008). Interactive "Video Doctor" counseling reduces drug and sexual risk behaviors among HIV-positive patients in diverse outpatient settings. PLoS One.

[3] Collins CB, Hearn KD, Whittier DN, Freeman A, Stallworth JD, Phields M (2010). Implementing packaged HIV-prevention interventions for HIV-positive individuals: considerations for clinic-based and community-based interventions. Public Health Rep.

[4] Govindasamy D, Ford N, Kranzer K (2012). Risk factors, barriers and facilitators for linkage to antiretroviral therapy care: a systematic review. AIDS.

[5] Kempf M, McLeod J, Boehme AK, Walcott MW, Wright L, Seal P, Norton WE, Schumacher JE, Mugavero M, Moneyham L (2010). A qualitative study of the barriers and facilitators to retention-in-care Among HIV-positive women in the rural southeastern united states: implications for targeted interventions. AIDS Patient Care STDs.

[6] Higa DH, Marks G, Crepaz N, Liau A, Lyles CM (2012). Interventions to improve retention in HIV primary care: a systematic review of U.S. studies. Curr HIV/AIDS Rep.

[7] Quanbeck A, Gustafson DH, Marsch LA, Chih M, Kornfield R, McTavish F, Johnson R, Brown RT, Mares M, Shah DV (2018). Implementing a mobile health system to integrate the treatment of addiction into primary care: a hybrid implementation-effectiveness study. J Med Internet Res.

[8] Kilbourne AM, Neumann MS, Pincus HA, Bauer MS, Stall R (2007). Implementing evidence-based interventions in health care: application of the replicating effective programs framework. Implement Sci.

[9] Claborn KR, Fernandez A, Wray T, Ramsey S (2015). Computer-based HIV adherence promotion interventions: a systematic review. Transl Behav Med.

[10] Bachmann LH, Grimley DM, Gao H, Aban I, Chen H, Raper JL, Saag MS, Rhodes SD, Hook EW (2013). Impact of a computer-assisted, provider-delivered intervention on sexual risk behaviors in HIV-positive men who have sex with men (MSM) in a primary care setting. AIDS Educ Prev.

[11] Hersch RK, Cook RF, Billings DW, Kaplan S, Murray D, Safren S, Goforth J, Spencer J (2013). Test of a web-based program to improve adherence to HIV medications. AIDS Behav.

[12] Kurth AE, Spielberg F, Cleland CM, Lambdin B, Bangsberg DR, Frick PA, Severynen AO, Clausen M, Norman RG, Lockhart D, Simoni JM, Holmes KK (2014). Computerized counseling reduces HIV-1 viral load and sexual transmission risk: findings from a randomized controlled trial. J Acquir Immune Defic Syndr.

[13] Noar SM, Black HG, Pierce LB (2009). Efficacy of computer technology-based HIV prevention interventions: a meta-analysis. AIDS.

[14] Fisher JD, Fisher WA (1992). Changing AIDS-risk behavior. Psychol Bull.

[15] Miller W, Rollnick S (2012). Motivational Interviewing. 3rd Edition.

[16] Prochaska JO, Velicer WF (1997). The transtheoretical model of health behavior change. Am J Health Promot.

[17] Harshbarger C, Taylor O, Uhrig J, Lewis M (2017). Positive health check: developing a web-based video counseling tool for HIV primary care clinics. J Comm Healthc.

[18] Hekler EB, Klasnja P, Riley WT, Buman MP, Huberty J, Rivera DE, Martin CA (2016). Agile science: creating useful products for behavior change in the real world. Transl Behav Med.

[19] Proctor E, Silmere H, Raghavan R, Hovmand P, Aarons G, Bunger A, Griffey R, Hensley M (2011). Outcomes for implementation research: conceptual distinctions, measurement challenges, and research agenda. Adm Policy Ment Health.

[20] Proctor EK, Landsverk J, Aarons G, Chambers D, Glisson C, Mittman B (2009). Implementation research in mental health services: an emerging science with conceptual, methodological, and training challenges. Adm Policy Ment Health.

[21] Sawesi S, Rashrash M, Phalakornkule K, Carpenter JS, Jones JF (2016). The impact of information technology on patient engagement and health behavior change: a systematic review of the literature. JMIR Med Inform.

[22] Yin R (2014). Case Study Research: Design and Methods.

[23] Thomas G (2011). How to Do Your Case Study: A Guide for Students and Researchers.

[24] Ritchie J, Spencer L, Bryman A, Burgess R (1994). Qualitative data analysis for applied policy research. Analysing Qualitative Data.

[25] Noar S, Harrington N (2012). eHealth Applications: Promising Strategies for Behavior Change.

[26] Aberg JA, Gallant JE, Ghanem KG, Emmanuel P, Zingman BS, Horberg MA, Infectious Diseases Society of America (2014). Primary care guidelines for the management of persons infected with HIV: 2013 update by the HIV medicine association of the Infectious Diseases Society of America. Clin Infect Dis.

[27] AIDSinfo: Clinical Guidelines Portal.

[28] Chen HT (2010). The bottom-up approach to integrative validity: a new perspective for program evaluation. Eval Program Plann.

[29] Glasgow RE, Lichtenstein E, Marcus AC (2003). Why don't we see more translation of health promotion research to practice? Rethinking the efficacy-to-effectiveness transition. Am J Public Health.

[30] Harshbarger CL, O'Donnell LN, Warner L, Margolis AD, Richardson DB, Novey SR, Glover LC, Klausner JD, Malotte CK, Rietmeijer CA (2012). Safe in the city: effective prevention interventions for human immunodeficiency virus and sexually transmitted infections. Am J Prev Med.

[31] Marley J (2000). Efficacy, effectiveness, efficiency. Aust Prescr.

[32] Neumann MS, O'Donnell L, Doval AS, Schillinger J, Blank S, Ortiz-Rios E, Garcia T, O'Donnell CR (2011). Effectiveness of the VOICES/VOCES sexually transmitted disease/human immunodeficiency virus prevention intervention when administered by health department staff: does it work in the "real world?". Sex Transm Dis.

